# New Immunotherapy Strategies in Breast Cancer

**DOI:** 10.3390/ijerph14010068

**Published:** 2017-01-12

**Authors:** Lin-Yu Yu, Jie Tang, Cong-Min Zhang, Wen-Jing Zeng, Han Yan, Mu-Peng Li, Xiao-Ping Chen

**Affiliations:** 1 Department of Clinical Pharmacology, Xiangya Hospital, Central South University, Changsha 410008, China; yulinhui19910530@163.com (L.-Y.Y.); jietang@csu.edu.cn (J.T.); ZhangCM126126@126.com (C.-M.Z.); wenjinganne@126.com (W.-J.Z.); yanhan501@126.com (H.Y.); elskesunny@163.com (M.-P.L.); 2 Institute of Clinical Pharmacology, Central South University, Hunan Key Laboratory of Pharmacogenetics, Changsha 410078, China

**Keywords:** breast cancer, immunotherapy, cancer vaccines, bispecific antibodies, immune checkpoint inhibitors, stimulatory molecule agonists

## Abstract

Breast cancer is the most commonly diagnosed cancer among women. Therapeutic treatments for breast cancer generally include surgery, chemotherapy, radiotherapy, endocrinotherapy and molecular targeted therapy. With the development of molecular biology, immunology and pharmacogenomics, immunotherapy becomes a promising new field in breast cancer therapies. In this review, we discussed recent progress in breast cancer immunotherapy, including cancer vaccines, bispecific antibodies, and immune checkpoint inhibitors. Several additional immunotherapy modalities in early stages of development are also highlighted. It is believed that these new immunotherapeutic strategies will ultimately change the current status of breast cancer therapies.

## 1. Introduction

Breast cancer is the most commonly diagnosed cancer and major cause of cancer death among women in less developed countries, with 882,900 cases diagnosed and 324,300 deaths in 2012, accounting for 25% of cancer cases and 15% of cancer deaths among females [1]. Risk factors for breast cancer include reproductive and endocrine factors such as the use of oral contraceptives, never having children, and a long menstrual history. Potentially modifiable risk factors include drinking, obesity, physical inactivity, and use of menopausal hormone therapy [2].

Recent studies classify breast cancer into four subtypes: Luminal A (ER^+^/PR^+^/HER2^−^, grade 1 or grade 2), Luminal B (ER^+^/PR^+^/HER2^+^, or ER^+^/PR^+^/HER2^−^ grade 3), HER2 overexpression (ER^−^/PR^−^/HER2^+^), and triple negative breast cancer (TNBC, ER^−^/PR^−^/HER2^−^). Luminal A subtype has a good prognosis and is sensitive to endocrine therapy, thus, general treatment can be endocrine therapy alone. Luminal B subtype is associated with high rate of tumor proliferation, among which HER2 negative Luminal B subtype is typically treated with endocrine therapy + chemotherapy; and HER2-positive Luminal B subtype is general treated with chemotherapy + anti-HER2 treatment + endocrine therapy. HER2 overexpression subtype features with poor prognosis and rapid progression, and the main recommended treatment is chemotherapy + anti-HER2 treatment. The negative expression of ER, PR and HER2 in TNBC has unique biological characteristics and strong heterogeneity, no standard treatment but chemotherapy is suggested for the subtype. Although recent progresses in early diagnosis and treatments have made breast cancer a treatable disease, multidrug resistance (MDR) remains the main obstacle in the treatment of metastatic breast cancer and the survival of patients with metastatic breast cancer is still 2–3 years [3,4].

**Figure 1:**
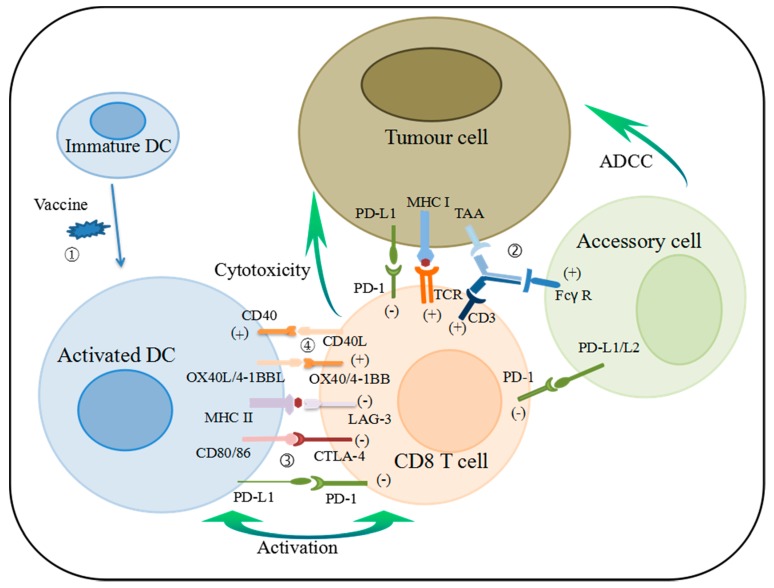
Schematic of action of selected immunotherapies in breast cancer. ① Vaccines initiate an immune response by providing target antigens to DCs and triggering their activation. Adaptive immune responses initiate at the lymph node through the interaction of T-cells and DCs. ② BsAbs can activate T-cell and accessory cell and cause tumor lysis by address TAA, CD3, and Fcγ-receptor of monocytes, macrophages, DCs, and NK cells simultaneously. ③, ④ Activated DCs present antigen as well as immune checkpoints and costimulatory molecules to T-cells. These molecules shape the quality and magnitude of the T-cell response. DCs, dendritic cells; BsAbs, bispecific antibodies; TAA, tumor-associated antigen; NK, nature killer; MHC I, major histocompatibility complex class I protein; ADCC, antibody dependent cellular cytotoxicity; Accessory cell, include monocytes, macrophages, dendritic cells, and NK cells.

Breast cancer is immunogenic, and multiple putative tumor-associated antigens (TAAs), such as HER-2 and Mucin 1 (MUC1), are observed in the cancer. These TAAs have been the successful targets of new drug development for cancer vaccine and bispecific antibody (bsAbs) over the past decade, some of which have been translated into tumor-specific immune responses and are proven to be clinically beneficial [5,6]. There is a growing body of evidence to support the fact that the immune cells in tumor microenvironment can effectively promote or inhibit tumor growth, which can be used as a prognostic indicator for breast cancer [7,8]. The immune cells in breast cancer tissue are mainly composed of T-lymphocytes (70%–80%), and the rest derived from B-lymphocytes amacrophages, natural killer cells and antigen-presenting cells (APCs) [9,10]. T-cells are activated by the recognition to tumor antigens submitted by APCs, and the strength and quality of the T-cell activation signals are connected with various receptor-ligand interactions [11] (Figure 1). 

First, the immune checkpoint signals of B7 family. CD28/Cytotoxic T-lymphocyte antigen-4 (CTLA-4) and B7-1 (CD80)/B7-2 (CD86) bind early in T-cell activation, and CD28 expressed on the initial or resting T-cells, while CTLA-4 expressed after T-cell activation with the affinity higher than CD28, play a role in promotion and inhibition of T-cells, respectively. Programmed cell death-1 (PDCD1, PD-1) signal, a new member of B7 family, can inhibit the immune response after T-cell activation. Second, programming signals: stimulatory molecules and cytokines. There is a tremendous body of evidence detailing the importance of cytokines in T-cell differentiation and fate determination [12,13,14,15]. Stimulatory molecules, such as CD40, OX40 and 4-1BB, are important in programming the flavor and longevity of T-cells responses necessary for the T-cells unique contribution to the inflammatory environment, including their cytolytic functionality and cytokine production that support neighboring T-cells and the humoral response [16]. Meanwhile, immune cells which are not present within the core also play specific roles in tumor process. Macrophages comprise a larger proportion of the stroma and influence reorganization of the extracellular matrix. There are three stages during the interaction of tumor cells and immune microenvironment: removal, balance, and escape [17]. The ultimate fate of the tumor cells includes either complete eradication or evolution of tumor cell variants that escape the immune surveillance and establish measurable tumors. These variants include loss of the expression of major histocompatibility complex (MHC) class I protein, inhibition in antigen processing and presentation pathways, mutation or loss of tumor antigens, deficiency in T-cell receptor (TCR) signaling and costimulation, and deficiency in cytokines synthesis [5].

In this review we discuss new findings in breast cancer immunotherapy, including recent achievements in immune checkpoint blockades and bispecific antibodies (bsAbs). We have also discussed therapeutic cancer vaccines and highlighted several additional immunotherapeutic modalities in early stages of development. In addition to these promising early results, information on 30 ongoing clinical trials that evaluate this class of immunotherapy in breast cancer are also summarized (Table 1).

## 2. Cancer Vaccines

Cancer vaccine belong to a class of biological response modifiers and generally contains an agent that resembles a TAA or a specific marker protein of tumor-causing microorganism. The agent stimulates the body’s immune system to recognize and fight against exactly tumor cells [18]. Adjuvant, an immunological or pharmacological agent that modifies the effect of other agents, is commonly used to boost immune response, particularly for cancer patients whose immune response to a simple vaccine may have weakened. It is an active field of research about the cancer vaccine. William Coley described the successful treatment of round-cell sarcoma with the intratumoral vaccination of *Streptococcus* and *Serratia* bacterial products since the in 1910s [19]. However, a successful vaccine should be able to stimulate the immune system as well as direct it towards a viable tumor target, or target a tumor antigen which plays a key role in the process of tumorigenesis and metastasis.

### 2.1. Antigen-Specific Vaccines

HER2 and MUC1 are two well-studied antigens in breast cancer. 25%–30% breast cancer patients exhibit HER2 overexpression and almost all breast cancers show MUC1 expression. The design of antigen specific vaccines can enlarge adaptive immune to a therapeutically beneficial level, for the levels of HER2 or MUC1 specific T-cells and antibodies are very low in most breast cancer patients [20,21].

#### 2.1.1. HER2-Derived Vaccines

Progresses have been made in the HER2-derived vaccines administered in the adjuvant settings. A dose schedule optimization phase I/II trial of the HER2-derived MHC class I peptide E75 with granulocyte-macrophage colony stimulating factor (GM-CSF) enrolled 195 HER2-positive breast cancer patients. The trial reported an improved 5-year disease-free survival (DFS) (89.7%) compared to GM-CSF-treated control groups (80.2%), while the local and systemic toxicities were mild [22]. AE37 is a HER2-derived MHC class II epitope targeting CD4^+^ T-lymphocytes which can elicit both CTL and CD4^+^ T_H_-cell responses. Result from a phase II trial that combined the AE37 peptide with GM-CSF for the adjuvant treatment of early stage breast cancers has shown similar toxicity profiles between vaccine group (AE37 + GM-CSF) and adjuvant group (GM-CSF), but a 40% reduction in recurrence was observed only in the vaccine-treated group at a median follow-up of 17 months [23].

Besides benefit from adjuvant therapy, the vaccines combined with HER2 monoclonal antibody or kinase inhibitor also obtained better curative effects. The University of Washington Tumor Vaccine Group found that combined therapy with trastuzumab (HER2 inhibitory antibody) and a HER2 vaccine boosted to greater levels of HER2-specific immune responses in patients with HER2 positive metastatic breast cancer than treated with trastuzumab alone, and the combination therapy was well tolerated [24]. It was well tolerated when HER2 vaccine was used in combination with lapatinib (tyrosine kinase inhibitor which interrupts the HER2 and epidermal growth factor receptor (EGFR) pathways) in trastuzumab-refractory breast cancers with HER2-overexpression, and anti-HER2-specific antibodies and HER2-specific T-cells were induced in 100% and 8% of patients respectively. However, there was no objective clinical responses [25]. These investigations suggest that the HER2-derived vaccines possess a promising prospect of research in breast cancer treatment, especially when combined with adjuvant or HER2 monoclonal antibody and kinase inhibitor, for the mild toxicity and well clinical responses.

#### 2.1.2. MUC1-Derived Vaccines

Mucin 1 (MUC1) is a member of the mucoprotein family and abnormally expressed in various epithelial cells and malignant tumors. MUC1 is overexpressed and aberrantly glycosylated in tumor cells, which contribute to the formation of epithelial cell carcinoma including breast cancer by promoting cell adhesion, blocking the apoptosis pathway and regulating intracellular growth signals [26]. MUC1 is the target of breast cancer early diagnosis biomarkers CA27-29 and CA15-3. Theratope (STn-KLH) is a therapeutic cancer vaccine that consists of a synthetic antigen including MUC1. In a phase III study involving 1208 patients with metastatic breast cancer treated with theratope concomitant endocrine, significantly longer time to progression (TTP) and overall survival (OS) than control group was observed, and this advantage is particularly pronounced in patients who have a robust antibody response to theratope. [27]. For the 12 breast cancer patients who were given monthly PANVAC vaccinations, a poxviral vaccine containing transgenes for MUC-1, CEA, and 3 T-cell costimulatory molecules, the side effects were some mild injection-site reactions, and 33% patients showed stable disease (SD) and 8% had a complete response (CR). Patients who had limited tumor burden, better CD4 response or higher number of CEA specific T-cells appeared to benefit from the vaccine [28]. 

L-BLP25 is a MUC1 antigen-specific vaccine. L-BLP25 vaccine in combination with letrozole could induce an antigen-specific immune response and increase the survival advantage obviously in MUC1-expressing breast cancer mouse model [29]. The PEGylated gold nanoparticle (AuNP)-based vaccine immobilizes chimeric peptides which consists of a glycopeptide sequence derived from MUC1 and the T-cell epitope P30 sequence, and this vaccine is able to significantly induce mice MHC-II mediated immune responses. Meanwhile, the antisera from AuNP treated mice can recognize human MCF-7 breast cancer cells [30]. Based upon these data, future trials evaluating the therapeutic effects of MUC1-derived vaccine in breast cancer are anticipated.

### 2.2. Cell-Based Vaccines

As vaccine responses are driven by APC, an effective approach to obtain the most effective APC is synthesis of dendritic cells (DCs) loaded with tumor antigen ex vivo and then administered to patients for immunotherapy. These vaccines present tumor antigens and activate tumor immunity directly or indirectly counting on the power of DCs [31]. 

Lapuleucel-T (APC8024) was prepared from peripheral-blood mononuclear cells (PBMC) and consisted of the sequences of HER-2 linked to granulocyte-macrophage colony-stimulating factor (GM-CSF). Lapuleucel-T was well tolerated in the clinical trial involving 18 lapuleucel-T treated patients with metastatic HER2^+^ breast cancer, without grade 3 or 4 adverse events. In addition, there was significant HER2-specific T-cell proliferation and 5.5% partial response (PR), 16.6% experienced SD lasting >1 years [32]. P53 serves as a favorable immunologic target as mutations are found in up to 30% of breast cancers. Spontaneous p53-reactive T-cells have been identified in more than 40% of patients with breast cancer-treated; In addition, the majority of breast cancer patients with high p53 expression have the ability to initiate p53-specific IFN-γ response [33]. In a phase II trial of a P53 DC vaccine involving 26 subjects with verified progressive breast cancer, among 19 patients continue treatment after 6 vaccinations weeks, 42% attained SD, indicating an efficacy of p53-specific immune therapy. The efficacy was associated with tumor p53 expression, p53 specific T-cells and serum YKL-40 and IL-6 levels [34]. Another clinical trial with P53 DC vaccine in combination with indoximod (IDO inhibitor) showed no effect, but it seems to benefit the subsequent salvage chemotherapy, and the causality is still in research (ASCO 2013 abstract 3069).

## 3. Bispecific Antibodies

Bispecific antibodies (bsAbs) contain specificities of two antibodies within a single molecule and address different antigens or epitopes simultaneously. At present, the majority of bsAbs for cancer immunotherapy were engineered to redirect immune effector cells to assemble and kill tumor cells. These bsAbs consist of one arm that binds a TAA on malignant cells and another arm that binds an activator receptor on immune effector cells, hence simultaneously togethering effector cells to the tumor and triggering their cytolytic activity for tumor killing [35].

Catumaxomab, a trifunctional antibody (triomab), is the first bsAb received market approval in 2009, and is used in the treatment of malignant ascites. It targets the EpCAM on tumor cells, and recruits T effector cells via binding to the CD3 subunit of the T-cell receptor complex, and could also activate monocytes, macrophages, dendritic cells, and NK cells at the same time by binding Fcγ-receptor [36]. In December of 2014, the second bsAb blinatumomab was approved for the therapy of patients with B cell acute lymphoblastic leukemia (BcellALL). Blinatumomab acts by binding CD3 and the CD19 antigen on ALL cells and features small size and lack of Fc region (BiTE) [37].

In light of these treatment application, bsAbs used for breast cancer immunotherapy are underway, even though almost in the stage of design or preclinical studies [38]. Ertumaxomab is an intact triomab targeting CD3 and HER2 simultaneously. The safety and antitumor efficacy was confirmed in preclinical studies and a phase I clinical trial in HER2 positive metastatic breast cancer patients. Most drug-related adverse events were mild, transient and reversible. The objective response rate (ORR) was 33% in 15 evaluable patients [6]. The ability of ertumaxomab to induce cytotoxicity against tumor cell lines with low HER2 antigen density, may provide a novel therapeutic option for breast cancer patients when trastuzumab treatment is inappropriate [39]. Recently, BsAb that armed activated T-cells (ATC) and expanded from leukapheresis product by IL2 and anti-CD3 monoclonal antibody, became a popular immune treatment model for research. In a phase I immunotherapy trial involving 23 women with metastatic breast cancer (MBC) treated with anti-CD3/anti-HER2 BsAb armed ATC along with low-dose IL-2 and GM-CSF, no dose-limiting toxicities was observed, 59.1% evaluable patients had SD or better. The median OS is 40 and 57.9 months for the HER2 0–2^+^ and HER2 3^+^ patients with SD, which were both improved since the median OS data prior to therapy are 27.4 and 57.4 months, respectively. At the same time, this treatment can induce both PBMC specific anti-SK-BR-3 and innate immune responses in women with MBC with a possible survival benefit [40]. However, further clinical trials are needed.

## 4. Immune Checkpoint Therapy

The genetic changes along with carcinogenesis provides a lot of immunogenic targets that can be recognized by the immune system [41]. Nevertheless, the functions of adaptive immune system are always inhibited by pathways that are dysregulated in tumors [42]. Immune checkpoints are cell surface molecules that play important physiologic roles in modulating immune response, preventing autoimmunity, and maintaining self-tolerance [43,44]. These surface receptors or ligands mediate immune inhibition in tumor microenvironment can result in suppression of the activation signal of original T-cells and infiltrating T-cells. Inhibitory antibodies targeting immune checkpoints have shown a great potential for the treatment of several solid cancers such as melanoma, bladder cancer, non–small cell lung cancer (NSCLC), and breast cancer [45,46,47,48]. Currently, immune checkpoint therapies by targeting CTLA-4, PD-1, or lymphocyte activation gene-3 (LAG-3) pathways for breast cancer are still in clinical trial.

### 4.1. CTLA-4 Inhibitors

Cytotoxic T-lymphocyte antigen-4 (CTLA-4) is the first immune checkpoint molecule shown to enhance antitumor immunity when inhibited [49]. It is known that two signal ways are required for T-cell activation: the first signal is the antigen recognition by TCR, and the second signal comes from the stimulus molecular combination of B7 and CD28. CTLA-4 is a CD28 homologue that binds to B7 with a higher affinity to out-compete the CD28 function and prevents T-cell from receiving a second signal [50]. CTLA-4 is upregulated after T-cell activation, providing a new insight into interfering with the inhibition of T-cell activation and antitumor therapy. 

CTLA-4 inhibitors caused a series of immune-related adverse reaction (irAEs) due to enhanced T-cell activation, which included hypophysitis, thyroiditis, colitis, and hepatitis. In addition, a considerable rate of irAEs was related to a significant rate of objective responses (OR) [11,51]. The mechanism may partially be explained by the reason that CTLA-4 is highly expressed on regulatory T-cells, which plays an important role in maintaining peripheral tolerance [52].

In addition to being FDA approved for melanoma, there are emerging trials regarding CTLA-4 inhibitors for breast cancer. Twenty-six patients with advanced, hormone-responsive breast cancer were given tremelimumab plus exemestane in a phase I clinical research [53]. This combination therapy was tolerable with the most common adverse reactions being diarrhea (46%) and pruritus (42%), and none developed grade 3 or 4 treatment-related diarrheas among 13 patients treated at the maximum tolerated dose (MTD). The trial reported a 42% SD lasting ≥12 weeks in patients utilizing this combination therapy, which was not worse than exemestane alone. The results showed no association between treatment efficacy and total counts or percentage of CD4/CD8 T-cells, but was associated with increased ICOS^+^ T-cells, which likely signals immune activation secondary to CTLA-4 inhibitor. Two other clinical trials about CTLA-4 inhibitor are ongoing: tremelimumab in combination with anti-B7H1 monoclonal antibody MEDI4736 in treatment of HER2 negative breast cancer (NCT02536794; Table 1) and ipilimumab in combination with anti-B7H3 monoclonal antibody MGA271 in treatment of TNBC (NCT02381314; Table 1). After all, further investigations are required to determine the CTLA-4 inhibitor safety profile and whether the outcome would be synergistic when used in combination with other agents or may accompanied by chemotherapy or radiation.

### 4.2. PD-1/PD-L1 Inhibitors

The programmed cell death-1 (PDCD1, PD-1) is expressed and remains upregulated during initial T-cell activation. The major ligand partners for PD-1 are PD-L1 (CD274 or B7-H1) and PD-L2 (CD273 or B7-DC). The PD-1 signal pathway has emerged as an interesting cancer therapeutic target [38]. Different from CTLA-4 that inhibits immune response at the initial T-cells activation step, PD-1 downregulates ongoing immunological effect at sites of reaction, that is, either in the periphery or in the neoplasm tissues [11]. PD1 is widely expressed on several immune cells including CD4^+^ and CD8^+^ T-cells, B cells, NK cells and T regulatory cells, and PD-L1 is a potential response marker for PD1/PD-L1 targeted therapies in major studies. 

PD-L1 protein expression is detected in 20%–30% breast cancer patients, especially in TNBC [54], while PD-L1 mRNA expression is detected in substantially larger subsets of breast tumors [55,56]. The clinical benefit of PD-1/PD-L1 inhibitor is associated with the expression level of PD-L1, though there is also study demonstrated clinical outcomes in PD-L1 negative tumors [57]. By contrast, PD-1/PD-L1 inhibitors showed lower incidence of irAEs, most grade 1 or 2. The most possible explanation may be that the PD-L1 expression is mostly limited to tumor and location of active inflammation which functions to down-modulate an immune response during the effector phase [58]. 

Nivolumab is the first anti-PD-1 antibody in clinical trials and shows a 31% objective response and median OS of 16.8 months in melanoma [59]. It is now approved for use in metastatic NSCLC and advanced melanoma. The existing studies in animal models [60,61,62] and clinic trials have shown that the PD-1/PD-L1 inhibitors also have potency for the treatment of breast cancer. In a non-randomized phase I study that enrolled 32 patients with PD-L1 positive recurrent/metastatic TNBC, the preliminary results showed that single agent pembrolizumab treatment is tolerated with 15.6% experienced at least one drug-related serious adverse event, and 16.1% of patients had a PR, 9.7% had SD [63], which was similar to the finding in PD-L1^+^/ER^+^/HER2^−^ metastatic breast cancer [64]. Two new PD-L1 inhibitors, atezolizumab and avelumab, are also under clinical trial. The efficacy of atezolizumab activity was assessed in 21 PD-L1-positive TNBC, with 24% objective responses (ORs): 10% showed complete response (CR) and 14% showed PR; 29% patients had progression-free survival of 24 weeks or longer. Such clinical efficacy is difficult to be achieved if these patients were treated with chemotherapy. However, several adverse reactions were also observed [40]. Combination of atezolizumab and nab-paclitaxel is tolerable with promising activity in patients with Metastatic TNBC [65]. Based on these preliminary results, a further Phase III study evaluated the combination therapy of atezolizumab and nab-paclitaxel in metastatic TNBC is ongoing (NCT02425891; Table 1). Another phase I study reported the clinical activity of avelumab in a cohort of patients with locally advanced or metastatic breast cancer. Avelumab have an acceptable safety profile with the most common were fatigue (19.6%), nausea (14.3%), and infusion-related reactions (11.9%), and there were 8.8% PRs in TNBC (five of 57), and all of the PD-L1^+^ PRs patients (four of 12) were TNBC [66]. It is meaningful to find an effective treatment with significant therapeutic activity in the heavily recurrent/metastatic TNBC, as most of these patients had received and progressed on multiple lines of therapy for advanced disease.

### 4.3. LAG-3 Target Therapy 

Lymphocyte activation gene-3 (LAG-3) is a receptor to MHC class II, which binds with higher affinity than CD4. The protein is expressed in the activated T-cells, NK cells and DCs. LAG-3 is reported to negatively regulate the activation, proliferation, and homeostasis of T-cells, in a similar fashion to CTLA-4 and PD-1, and also plays a role in Tregs suppressive function [67]. LAGs are known to be involved in the maturation and activation of DCs. IMP321 is a soluble form of LAG-3 that functions as an APC active agent. A Phase I/II study has evaluated the effect of combined therapy with paclitaxel plus IMP321: patients with metastatic breast carcinoma were administered IMP321 every 2 weeks subcutaneously with weekly 80 mg/m^2^ intravenous paclitaxel. The combined therapy induced both a sustained increase in the number of activated APC and an increase in the percentage of NK and long-lived cytotoxic effector-memory CD8 T-cells. The trail reported 50% ORR and 90% clinical benefit in 6 months in patients utilizing combined therapy compared to 25% ORR and <50% clinical benefit in the historical control group. No clinically significant IMP321-related adverse events were reported [68]. A phase II trial with combination of IMP321, placebo, and paclitaxel is ongoing (NCT02614833; Table 1). 

## 5. Stimulatory Molecule Agonist Antibodies

Besides immune checkpoints providing a negative signal to T-cells activation, a number of positive molecules, whose engagement upregulates T-cell function. The eventual outcome of T-cell activating interaction involves an integration of both the negative and positive signals present during that interaction. Though it is still in early stages of research, stimulatory molecule agonist antibodies have enormous potential in breast cancer and are introduced here with an eye on future development.

### 5.1. OX40 Agonist Antibodies

OX40 (CD134) is a member of the TNF receptor superfamily, a stimulatory molecule that are expressed in activated immune cells and tumor infiltrating lymphocytes (TILs) in breast cancer, and the expression can be increased gradually as soon as T-cells identify their specific antigens [69]. The engagement between OX40 on a T-cell and OX40 ligand on an APC not only provides a powerful stimulatory signal to the T-cells, but also provides an inhibitory signal to Tregs [70]. Based on preclinical cancer models that showed potent antitumor activity against multiple tumor types of anti-OX40 antibody, which is dependent on both CD4^+^ and CD8^+^ T-cells [71,72,73,74], a phase I study showed that OX40 agonist antibody (9B12) induced the regression of at least one metastatic lesion in 40% (12/30) patients with advanced cancer with acceptable toxicity (most toxicity was grade 1 or 2). Addition to clinical effects, the immunologic effects were increased including proliferation of circulation CD4^+^ and CD8^+^ T-cells, responses to recall and naive reporter antigens, and endogenous tumor-specific immune responses [75]. A second anti-OX40 antibody (MEDI6469) is now undergoing phase I/II clinical trials in combination with stereotactic radiotherapy in progressive metastatic breast cancer (NCT01862900). Base on immunologic effects of anti-OX40, it is rationale for application in conjunction with other therapy agent, such as vaccination, to increase T- and B-cell responses to immunize antigens in future clinical studies.

### 5.2. 4-1BB Agonist Antibodies

4-1BB is also a member of the TNF receptor superfamily which can be induced on diverse immune cell populations following activation, such as T-cells, NK cells, regulatory T-cells, and NK T-cells (NKT) [76]. The engagement of 4-1BB and 4-1BBL, which are predominantly expressed by activated APCs can induce an activating signal in CD8^+^ T-cells and NK cells, resulting in increased pro-inflammatory cytokine secretion, cytolytic activity, and antibody-dependent cell-mediated cytotoxicity (ADCC) [77,78]. Urelumab (BMS-663513) is a monoclonal antibody specific for 4-1BB during phase II testing in melanoma [79]. As 4-1BB agonistic antibody stimulates the activation of NK cells which may enhance NK cytotoxic function, there is an alternative approach aiming at enhancing ADCC by stimulation of NK cells with an anti-4-1BB agonistic antibody. Results have shown that antibodies targeting 4-1BB synergized with trastuzumab can kill tumor cells more efficiently in murine xenotransplant models of human breast cancer [80]. 4-1BB serves as a potential vaccine target in breast cancer, but has yet to be evaluated in humans.

### 5.3. CD40 Agonist Antibodies

CD40 is also a member of the TNF receptor superfamily and a stimulatory protein found on APCs and is required for their activation. The CD40 ligation plays a role in APCs stimulation and maturation leading to an increase in antigen presentation and cytokine production, and a subsequent increase in the activation of antigen specific T-cells [81]. The first CD40 agonist monoclonal antibody observed in patients with solid tumors is CP-870,893. In a phase I study in patients with stage III and IV solid malignancies, CP-870,893 was well tolerated, and 27% of melanoma patients had a partial response [82]. Kawaguchi et al. suggested that mRNA expressions of CD40 in PBMCs are affected by breast cancer disease progression [83]. CD40 stimulation by its soluble recombinant human CD40 ligand directly inhibits human breast cancer cells in vitro and in SCID mice model [84]. A clinical study reported a significant difference in expression of cytoplasmic CD40 between breast cancer subtypes, and cytoplasmic expression of CD40 is related to a better prognosis [85], which suggest that CD40 may have potential as a new prognostic factor in breast cancer.

## 6. Conclusions

Breast cancer can be immunogenic, and the tumor immune microenvironment induced several local immune responses in the tumor tissue. In addition to immune environment, the tumor itself, such as the type of breast tumor, the area within the tumor and microenvironment also influences tumor process. For example, each subtype of breast cancer has a distinct prognosis and natural history. Poor chemotherapy prognostic factors including lack of ER and PR expression, high tumor grade, and lymph node involvement, are associated with a significantly higher CD3^+^, CD8^+^ and FoxP3^+^ cellular infiltrate. The immunotherapy effect can be influenced by immune cells intratumoral and peritumoral distribution, immune cells composition, and the breast tumor overall immune context and histology. The ultimate responses either suppress tumor growth or completely eliminate tumors through immune-mediated cell death, or promote tumor progression by providing inflammatory environment for tumor cells survive immunosurveillance. Nevertheless, Clinical benefits from immunotherapy for breast cancer have been shown in a growing number of trials (Table 2).

Current immunotherapy is still in its infancy for breast cancer. As mentioned above, it cannot be denied that a portion of breast cancer patients can’t benefit from these immunotherapy strategies. Despite the clinical benefit of PD-1/PD-L1 inhibitor is affected by the expression level of PD-L1, the majority of patients (76%) with PD-L1-positive TNBC were completely refractory to atezolizumab therapy [45], which indicated interindividual difference in drug response. As a heterogeneous disease, breast cancer is characterized by diversified molecular phenotypes that correlate with different drug resistant and treatment outcomes. For example, genetic polymorphisms of *CYP1B1* and *ABCB1* are associated with the clinical response to chemotherapy in breast cancer [86]. Since breast cancer is also an immunogenic disease, part of the patients does benefit from immunotherapy, yet it historically has been resistant to immunotherapy. With the development of molecular biology and genomics, pharmacogenomics has been shown to be effective in a significant proportion of cancer therapies in predicting therapeutic responses. The polymorphism number and site in *Ribonucleotide reductase *(*RNR*) showed significant correlation with leukopenia in heavily metastatic breast cancer patients with gemcitabine monotherapy [87]. Breast cancer patients with low *COX-2* expression and *PIK3CA* wild type tumors had worse DFS compared to all other subgroups in celecoxib treatment, which suggest *COX-2* expression and *PIK3CA* mutation may be good prognostic and predictive biomarkers for celecoxib therapy [88]. *CD95* and *MBL2*, two immune function genes, the promoter single nucleotide polymorphisms (SNPs) of *CD95* (rs2234767) and *MBL2* (rs7096206) are associated with grade 3 infection following treatment of breast cancer with cytotoxic therapy [89]. Polymorphisms in immune function genes may provide means for predicting clinical benefit or toxicity from immunotherapy. Further pharmacogenomics studies focused on immune system should be performed to elucidate the mechanisms and identify biomarker signatures in patients with higher toxicity and with resistant or responsive outcome, before the administration of immunotherapy strategies. Accordingly, pharmacogenomics may greatly improve the efficacy of immunotherapy for breast cancer as well as other cancer, and reduce the incidence of adverse reactions. Our current study revealed difference in the cytotoxicity to HER2 positive breast cancer cells when trastuzumab was incubated with PBMCs from different healthy individuals, and several polymorphisms in immune function genes have been found related to this difference (data not shown). Based on pharmacogenomics studies, these immunotherapy strategies may achieve a great success in personalized therapy and ultimately improve the current status of therapy for breast cancer patients.

## Figures and Tables

**Table 1:** Examples of ongoing clinical trial of immunotherapy in breast cancer.

Trial ID	Phase	Breast Cancer Subtype	Primary Endpoint	Study
**Vaccines**				
NCT02427581	I	TNBC	Safety	Poly ICLC
NCT01730118	I	HER2^+^ BC	Cardiac toxicity and anti-HER2 antibody concentration	Autologous Ad HER2 dendritic cell vaccine
NCT02018458	I/II	phase1: LA TNBC; phase 2: ER^+^/HER2^−^ BC	Safety	DC vaccination + Preoperative chemotherapy
NCT01570036	II	HER2^+^ BC	DFS	E75 + Trastuzumab
NCT02061332	I/II	BC	Blood pressure, temperature, pulse	HER2 Pulsed Dendritic Cell Vaccine
NCT01376505	I	BC	Immune response and clinical benefit	HER2 vaccine
NCT02140996	I	BC	Safety and tolerability	Ad-sig-hMUC-1/ecdCD40L vector vaccine
**BsAbs**				
NCT01730612	I/II	HER2^−^CEA^+^ BC	Tumor targeting and signal/noise ratio	TF2 + 68 Ga-IMP-288
**CTLA-4**				
NCT02536794	II	HER2^−^ BC	Response rate	MEDI4736 + Tremelimumab
NCT02381314	I	TNBC	Adverse event	MGA271 + Ipilimumab
**PD-1**				
NCT02661100	I/II	Advanced TNBC	DLT	Pembrolizumab + CDX-1401 + Poly ICLC
NCT02453620	I	HER2^−^ BC	Adverse event	Entinostat + Ipilimumab + Nivolumab
NCT02129556	I/II	HER2^+^BC (Trastuzumab-resistant)	DLT	Pembrolizumab
NCT02309177	I	BC	DLT, Safety, Grade 3 or 4 TEAE	Nab-Paclitaxel + Nivolumab + Gemcitabine + Carboplatin
NCT02404441	I/II	TNBC	DLT and ORR	PDR001
NCT02555657	III	TNBC	PFS and OS	Pembrolizumab + Capecitabine + Eribulin + Gemcitaine + Vinorelbine
**PD-L1**				
NCT02643303	I/II	BC	Phase 1 Safety and tolerability Phase 2 ORR, PFS, OS	Durvalumab + Tremelimumab + Poly ICLC
NCT02628132	I/II	TNBC	Adverse event	Paclitaxel + Durvalumab
NCT02685059	II	TNBC	pCR	Durvalumab + Placebo + nab-Paclitaxel + Epirubicin + Cyclophosphamide
NCT02725489	II/III	TNBC	Tolerability and adverse event	Vigil™ + Durvalumab
NCT02425891	III	Metastatic BC/TNBC	PFS and OS	Atezolizumab + Nab-Paclitaxel + Placebo
NCT02478099	II	TNBC	ORR	Atezolizumab
NCT02649686	I	HER2^+^ Metastatic BC	Confirm phase II dose	Durvalumab + Trastuzumab
NCT02708680	I/II	Advanced TNBC	DLT, MTD and PFS	Entinostat + Atezolizumab
**LAG-3**				
NCT02614833	II	Stage IV BC	PFS	IMP321 + Placebo + Paclitaxel
**OX40**				
NCT01862900	I/II	Metastatic BC	DLT and safety profile	MEDI6469
**4-1BB**				
NCT02554812	II	TNBC	DLT and ORR	PF-05082566 + Avelumab

Notes: Poly ICLC, CDX-1401 (peptide vaccine); E75 (HER2 vaccine); trastuzumab (HER2 inhibitor); TF2 (anti-CEA X anti-HSG BsAbs); 68 Ga-IMP-288 (peptide IMP-288 radiolabeled with gallium-68); MGA271 (B7H3 inhibitor); tremelimumab, ipilimumab (CTLA-4 inhibitor); pembrolizumab, nivolumab, PDR001 (PD-1 inhibitor); entinostat (benzamide histone deacetylase inhibitor); MEDI4736, durvalumab, atezolizumab, avelumab (PD-L1 inhibitor); Vigil™ (autologous tumor cell vaccine); IMP321 (soluble form of LAG-3), MEDI6469 (OX40 agonist); PF-05082566 (4-1BB agonist); nab-paclitaxel, gemcitabine, carboplatin, capecitabine, eribulin, vinorelbine, paclitaxel, epirubicin, cyclophosphamide (chemotherapy medication). Abbreviations: BC, breast cancer; TNBC, triple negative breast cancer; LA TNBC, locoregional advanced triple-negative breast cancer; BsAbs, bispecific antibodies; DFS, Disease-free survival; DLT, dose limiting toxicity; TEAE, treatment emergent adverse events; ORR, objective response rate; PFS, progression-free survival; OS, overall survival; pCR, pathological complete response; MTD, maximum tolerated dose.

**Table 2:** Examples of completed clinical trials of immunotherapy in breast cancer.

Phase	Breast Cancer Subtype	*n*	Study	Immune-Related Response	Clinical Benefit	Reference
**Vaccines**						
I/II	HER2^+^ BC	195	E75 + GM-CSF	All patients developed a DTH response to E75 after vaccination, and that DTH reactions were dose dependent	Toxicities were mild; improved 5-year DFS	[22]
II	Early stage BC	206	AE37 + GM-CSF	Increase in DTH response to AE37, decrease in CD4^+^CD25^high^ CD127^low^ regulatory T-cells	A reduction in recurrence	[23]
I/II	Stage IV HER2^+^ MBC	22	HER2 vaccine + Trastuzumab	Increase the HER2-specific immune responses	Well tolerated	[24]
I	HER2^+^ BC (trastuzumab-refractory)	12	HER2 vaccine + Lapatinib	Anti-HER2-specific antibodies and HER2-specific T-cells were induced in 100% and 8% of patients respectively	Well tolerated; no objective clinical responses	[25]
III	MBC	1208	Theratope + Endocrine	Antibody response to theratope	Longer TTP and OS than control group	[27]
I	MBC	12	PANVAC	Limited tumor burden, better CD4 response or higher number of CEA specific T-cells appeared to benefit from this vaccine	33% SD and 8% CR	[28]
I	HER2^+^ MBC	18	Lapuleucel-T	Significant HER2-specific T-cell proliferation	Without grade 3 or 4 adverse events; 5.5% PR, 16.6% experienced SD lasting >1 years	[32]
II	MBC	26	P53 DC vaccine	The efficacy was associated with tumor p53 expression, p53 specific T-cells and serum YKL-40 and IL-6 levels	8/19 evaluable patients attained SD	[34]
**BsAbs**						
I	HER2^+^ MBC	15	Ertumaxomab	A strong T helper cell type 1-associated immune response	Most drug-related adverse events were mild; The ORR was 33%	[6,39]
I	MBC	23	Anti-CD3/anti-HER2 BsAb armed ATC along with low-dose IL-2 and GM-CSF	Induce both PBMC specific anti-SK-BR-3 and innate immune responses	No dose-limiting toxicities was observed; 59.1% evaluable patients had SD or better, and the median OS was 36.2 months	[41]
**CTLA-4 **						
I	MBC	26	Tremelimumab + Exemestane	Treatment was associated with increased peripheral CD4^+^ and CD8^+^ T-cells expressing ICOS and a marked increase in the ratio of ICOS^+^ T-cells to FoxP3^+^ regulatory T-cells.	Tolerable, and 42% patients experienced SD lasting ≥12 weeks.	[54]
**PD-1/PD-L1 **						
I	PD-L1^+^ mTNBC	32	Pembrolizumab	NR	15.6% experienced at least one drug-related serious adverse event; 16.1% PR, 9.7% SD	[64]
I	PD-L1^+^ TNBC	21	Atezolizumab	Treatment was associated with increased plasma cytokine concentrations and proliferating CD8 cells	24% ORs, 29% patients had PFS of 24 weeks or longer; several adverse reactions	[46]
I	mTNBC	11	Atezolizumab + Nab-paclitaxel	NR	Tolerable, 4 PRs and 1 SD	[66]
I	Locally MBC	168	Avelumab	NR	Among all patients with PD-L1 expressing, 33.3% (4 of 12) had PRs.	[67]
**LAG-3 **						
I/II	MBC	30	IMP321 + paclitaxel	Increase the number of activated APC, percentage of NK and long-lived cytotoxic effector-memory CD8 T-cells	ORR was 50%, and clinical benefit was noted in 90% in 6 months with no clinically significant IMP321-related adverse events	[69]
**OX40 **						
I	Advanced cancer (refractory to conventional therapy)	30	9B12	Immunologic effects were increased including proliferation of circulation CD4 and CD8 T-cells, responses to recall and naive reporter antigens, and endogenous tumor-specific immune responses	Induced the regression of at least one metastatic lesion in 40% patients	[76]

BC, breast Cancer; MBC, metastatic breast cancer; TNBC, triple negative breast cancer; mTNBC, metastatic triple-negative breast cancer; GM-CSF, granulocyte-macrophage colony stimulating factor; BsAbs, bispecific antibodies; DTH, Delayed-type hypersensitivity; ICOS, inducible T-cell costimulator; DFS, disease-free survival; TTP, time to progression; OS, overall survival; SD, stable disease; CR, complete response; PR, partial response; ORR, objective response rate; ORs, objective responses; PFS, progression-free survival; NR, not reported.
